# Self-Seeded MOCVD Growth and Dramatically Enhanced Photoluminescence of InGaAs/InP Core–Shell Nanowires

**DOI:** 10.1186/s11671-018-2690-3

**Published:** 2018-09-05

**Authors:** Xianghai Ji, Xiren Chen, Xiaoguang Yang, Xingwang Zhang, Jun Shao, Tao Yang

**Affiliations:** 1 Key Laboratory of Semiconductor Materials Science, Institute of Semiconductors, Chinese Academy of Sciences, Beijing, 100083 People’s Republic of China; 2 College of Materials Science and Opto-Electronic Technology, University of Chinese Academy of Sciences, Beijing, 100049 People’s Republic of China; 3 National Laboratory for Infrared Physics, Shanghai Institute of Technical Physics, Chinese Academy of Sciences, Shanghai, 200083 People’s Republic of China

**Keywords:** Core−shell nanowire, InGaAs/InP, Metal−organic chemical vapor deposition, Optics properties

## Abstract

We report on the growth and characterization of InGaAs/InP core–shell nanowires on Si–(111) substrates by metal-organic chemical vapor deposition (MOCVD). The strain at the core–shell interface induced by the large lattice mismatch between the InGaAs core and InP shell materials has strong influence on the growth behavior of the InP shell, leading to the asymmetric growth of InP shell around the InGaAs core and even to the bending of the nanowires. Transmission electron microscopy (TEM) measurements reveal that the InP shell is coherent with the InGaAs core without any misfit dislocations. Furthermore, photoluminescence (PL) measurements at 77 K show that the PL peak intensity from the InGaAs/InP core−shell nanowires displays a ∼ 100 times enhancement compared to the only InGaAs core sample without InP shell due to the passivation of surface states and effective carrier confinement resulting from InP shell layer. The results obtained here further our understanding of the growth behavior of strained core–shell heterostructure nanowires and may open new possibilities for applications in InGaAs/InP heterostructure nanowire-based optoelectronic devices on Si platform.

## Background

III–V semiconductor nanowires have been recognized as promising candidates for next-generation nanoscale devices owing to their unique electronic, optical, and geometrical properties [1–4]. Among the III–V semiconductor materials, ternary InGaAs nanowire is extremely attractive for photonics and optoelectronic applications due to its excellent physical properties, such as large controllable range of direct band-gap, small carrier effective mass, and high carrier mobility. In addition, the integration of III–V materials with a Si platform, which enables the combination of the advantages of the unique physical properties of III–V materials with mature complementary metal oxide semiconductor (CMOS) technology, has been intensely studied. Because of the small footprint, nanowires provide an opportunity for the integration of III–V materials with Si ignoring the large difference in lattice parameters between the materials [5, 6]. Thus far, various devices based on ternary InGaAs nanowires have been fabricated on Si substrates, including low-power high-speed transistors [7, 8], tunneling-based devices [9, 10], light-emitting diodes (LEDs) [11], photonic devices [12, 13], and solar cells [14, 15].

However, due to the high surface-to-volume ratio of the one-dimensional nanowire, the numerous surface states have become a main limitation in achieving high-performance nanowire-based optoelectronic devices. On the one hand, these surface states can greatly degrade both the electronic and the optical properties of the III–V materials through scattering and a non-radiative recombination process [16–20]. On the other hand, for nanowires of some narrow-gap materials (such as InAs, In−rich InGaAs), the high density of surface states can lead to a bend of the electronic band structure near the nanowire surface (surface Fermi level pinned effect). Such a non-flat band structure will further cause charge carrier redistribution, which can strongly hinder the performance of optical nanowire-based devices [21]. Therefore, eliminating these surface states is highly necessary. For ternary InGaAs nanowires with higher In composition, InP is a desirable surface passivating layer, as the material system forms a type I band-gap alignment, which can confine carriers in InGaAs effectively. Furthermore, for InGaAs/InP material system, which has been widely investigated in planar structures, its emission wavelength is tunable in the range of 1.31–1.55 μm, which has a promising prospect in optical fiber communication.

In this work, we carried out the growth and characterization of InGaAs/InP core–shell nanowires on Si–(111) substrates using metal-organic chemical vapor deposition (MOCVD). It is found that the strain at the core–shell interface resulting from large lattice mismatch between the core and shell materials has strong influence on the growth behavior of the InP shell. The large lattice mismatch between the core and shell materials can lead to non-uniform nucleation of InP coating layer around the InGaAs core nanowires and even to the bending of the nanowires. By optimizing growth conditions, the InGaAs/InP core−shell nanowires with good morphology can be achieved. Moreover, photoluminescence (PL) measurements at 77 K show that PL peak intensity from the InGaAs/InP core−shell nanowires shows an about 100 times enhancement compared to the bare InGaAs nanowires due to the passivation of surface states and effective carrier confinement via InP coating layer.

## Methods/Experimental

### Nanowire Growth

The InGaAs/InP core−shell nanowires were grown by a close-coupled shower head MOCVD system (AIXTRON Ltd., Germany) at 133 mbar. Trimethylindium (TMIn) and trimethylgallium (TMGa) were used as group III precursors, and arsine (AsH_3_) and phosphine (PH_3_) were used as group V precursors. Ultra-high-purity hydrogen (H_2_) was used as a carrier gas, and the total flow rate of H_2_ was 12 slm. Prior to growth, the Si–(111) substrates were heated to 635 °C for annealing and then cooled to 400 °C under AsH_3_ flux to form (111)B-like surfaces [22]. The InGaAs core nanowires were grown at 565 °C for 15 min. During the growth process, TMIn and AsH_3_ flow rates are of 0.8 × 10^− 6^ mol/min and 1.0 × 10^− 4^ mol/min, while TMGa flow rate is varied. The TMGa vapor-phase composition, Xv, which was defined as the ratio of the flow rates TMGa/(TMGa+TMIn), was varied from 30 to 40%. The InP shell was grown at 565 °C for 10 min with TMIn and PH_3_ flow rates of 2 × 10^− 6^ mol/min and 8.0 × 10^− 4^ mol/min, respectively. After growth, the samples were cooled to room temperature using PH_3_ as a protective agent.

### Characterization Methods

The morphology of the nanowires was characterized by scanning electron microscopy (SEM) (Nova Nano SEM 650) and transmission electron microscopy (TEM) (JEM2010F TEM; 200 kV) in conjunction with X-ray energy-dispersive spectroscopy (EDS) was used to investigate the crystal structure and composition, respectively. For TEM observations, the nanowires were mechanically transferred from the samples to copper grids coated with a carbon film. To investigate the optical properties of the grown nanowires, photoluminescence (PL) measurements were performed using a 532 nm wavelength laser as the excitation source. The samples were excited with a laser power of ~ 100 mW over a spot-size with a diameter of approximately 150 μm. PL signal was directly fed into a Fourier transform infrared (FTIR) spectrometer and recorded by a liquid nitrogen-cooled InSb detector. The moving mirror in FTIR spectrometer ran in a rapid-scan mode [23], differing from the step-scan modulated PL measurements on InAs nanowires in mid-infrared region [24].

## Results and Discussion

**Fig. 1:**
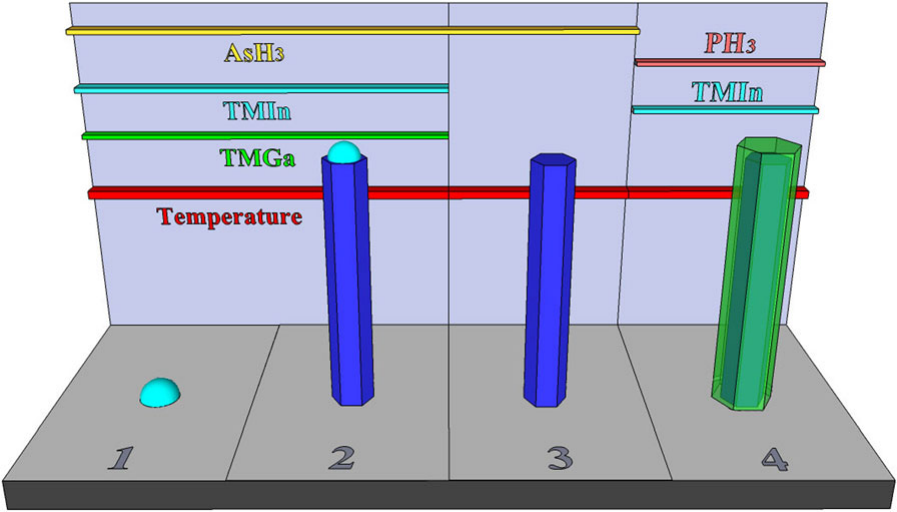
Schematic illustration of the growth of InGaAs/InP core−shell nanowires and the source-supply sequences for the nanowire growth

Figure 1 shows a schematic illustration of the growth of InGaAs/InP core−shell nanowires on a Si–(111) substrate and the source−supply sequences for the growth of the nanowires. InGaAs nanowires grow by a self-catalyzed mechanism [25]. Please note that, In droplets will be consumed under a AsH_3_ atmosphere (shown in region 3 in Fig. 1). The overgrowth of InP shell was initiated by switching the AsH_3_ to PH_3_ flux and opening the TMIn flux simultaneously.

**Fig. 2:**
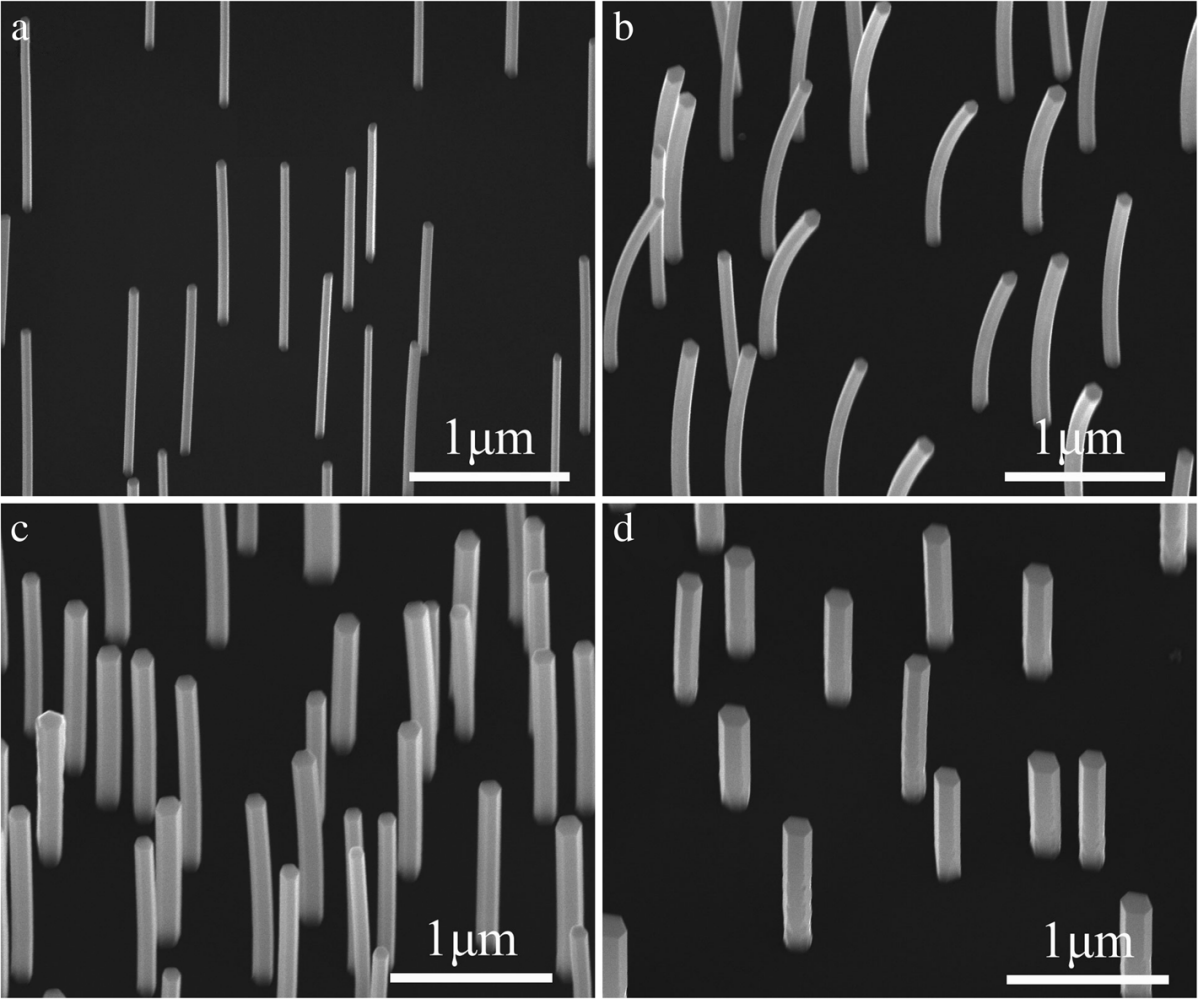
**a** 30° -tilted SEM images of the InGaAs nanowires, and InGaAs/InP core−shell nanowires with Xv, of **b** 30%, **c** 35%, and **d** 40%

Figure 2a, b shows typical SEM images of bare InGaAs and InGaAs/InP core−shell nanowires with Xv = 30%, respectively. All InGaAs nanowires are vertically aligned on the Si substrate with uniform diameter along the entire length. After the subsequent growth of InP shell, the nanowires are still with smooth side-facets, indicating the optimization of growth parameters. From the statistical distributions of diameters of the bare InGaAs and InGaAs/InP core−shell nanowires, the average diameter of the nanowires increases from ∼ 65 to ∼ 95 nm after the growth of InP shell, which indicates the average InP shell thickness of approximately 15 nm. However, the InGaAs/InP core−shell nanowires in Fig. 2b are visibly bent, which is induced by the stress on the InGaAs core nanowire caused by the InP shell due to the large lattice mismatch between the core and shell materials. Figure 2c, d shows SEM images of the InGaAs/InP core−shell nanowires with Xv of 35%, and 40%, respectively. Compared with the nanowires in Fig. 2b, the bending of InGaAs/InP core−shell nanowires with Xv of 35% reduced greatly (Fig. 2c). Further increasing the Xv to 40%, the nanowires are straight without visible bending (Fig. 2d). This phenomenon can be ascribed to the reduction in the lattice mismatch between the InGaAs core and InP shell materials with the increase of Ga composition. In addition, from the statistical distributions of the diameters of InGaAs/InP core−shell nanowires, as the Ga composition increases, the diameter of nanowire increases at the same time, which can also hinder the InGaAs core nanowires from bending after the InP coating.

**Fig. 3:**
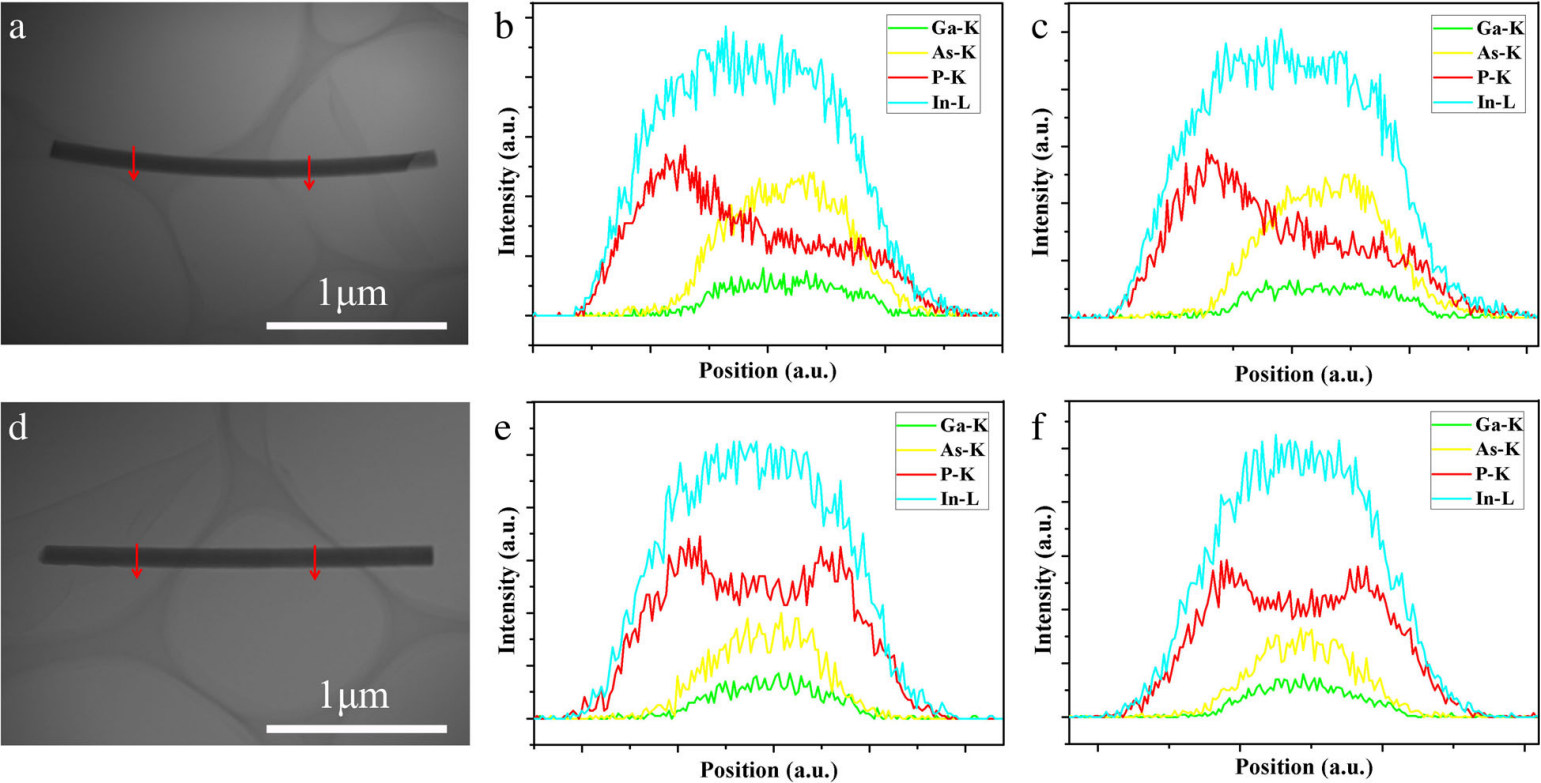
**a** HRTEM image of the bare InGaAs nanowire (Xv = 35%) acquired from the < 110> zone axis. The inset is the corresponding selected area electron diffraction (SAED) pattern. **b** Low-magnification TEM image of an InGaAs/InP core−shell nanowire (Xv = 35%). **c** HRTEM image of the nanowire viewed from the < 110> zone axis. The red dashed line indicates the interface between the core and the shell

To investigate the crystal structure of the grown nanowires and confirm the existence of the core–shell structure after growing the InP shell, detailed TEM measurements were conducted. As shown in Fig. 3a, the crystal structure of the InGaAs nanowire with Xv of 35% is composed of a polytype of wurtzite (WZ) and zinc-blende (ZB) structures with a large number of stacking faults (SFs) along its growth direction, and due to the coexistence of WZ and ZB structures along with structural defects, the corresponding selected area electron diffraction (SAED) spots split and are slightly elongated along the growth direction (inset in Fig. 3a). These planar defects are commonly observed in the growth of InAs or InGaAs nanowires without foreign catalyst by MOCVD [26–28]. Figure 3b shows a bright-field (BF) low-resolution TEM image of a typical InGaAs/InP core−shell nanowire with Xv of 35% (as shown in Fig. 2c). After InP coating, the nanowire is still quite straight without tapering. A corresponding high-resolution (HR) TEM image is shown in Fig. 3c. A clear interface between InGaAs core and InP shell can be observed. In addition, no misfit dislocations were found by following the {111} planes at the core−shell interface. Therefore, the as−grown InP shell is coherent with the InGaAs core. Moreover, due to the coherently epitaxial growth of the InP coating layer, the crystal structure of the InP shell will completely inherit that of the InGaAs core nanowire, as confirmed by the mixed WZ/ZB structure of InGaAs/InP core−shell nanowire in Fig. 3c. This phenomenon has been observed in core–shell nanowires of other material systems [29–31], and the behavior highlights the need to improve the crystal quality of the self-catalyzed InGaAs nanowires.

**Fig. 4:**
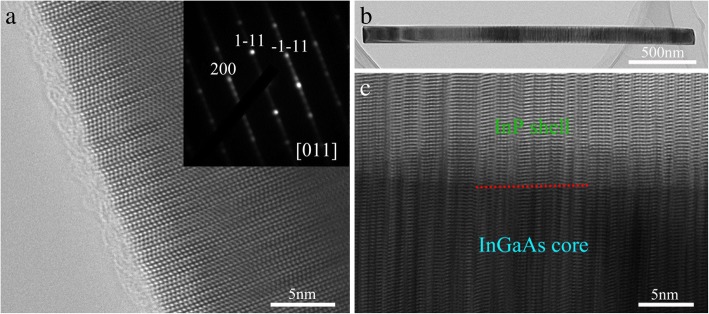
**a** A low-magnification TEM image of an InGaAs/InP (Xv = 30%) core–shell nanowire. **b**, **c** EDS line scans along the two red lines marked in **a**. **d** A low-magnification TEM image of an InGaAs/InP (Xv = 35%) core–shell nanowire. **e**, **f** EDS line scans along the two red lines marked in (**d**)

Figure 4a–c shows a low-magnification TEM image and EDS analyses of a typical InGaAs/InP core–shell nanowire shown in Fig. 2b. According to the EDS line scans across the nanowire, P signal can be obviously identified in the spectra, indicating the existence of InP shell around InGaAs core. Whereas, the EDS spectrum of P signal is asymmetric, which implies that the overgrowth of InP shell is non-uniform around the InGaAs core nanowire. We speculate that this phenomenon may be mainly induced by the relatively large lattice mismatch between core and shell materials, and such non-uniform nucleation of InP shell will further result in the bending of the nanowires. In contrast, for the straight InGaAs/InP (Xv = 35%) core–shell nanowire in Fig. 2c, EDS analyses in Fig. 4e–f show symmetric distributions of P signal throughout the nanowire, indicating the improved uniformity of InP shell around the InGaAs core with the increase of Ga content here.

**Fig. 5:**
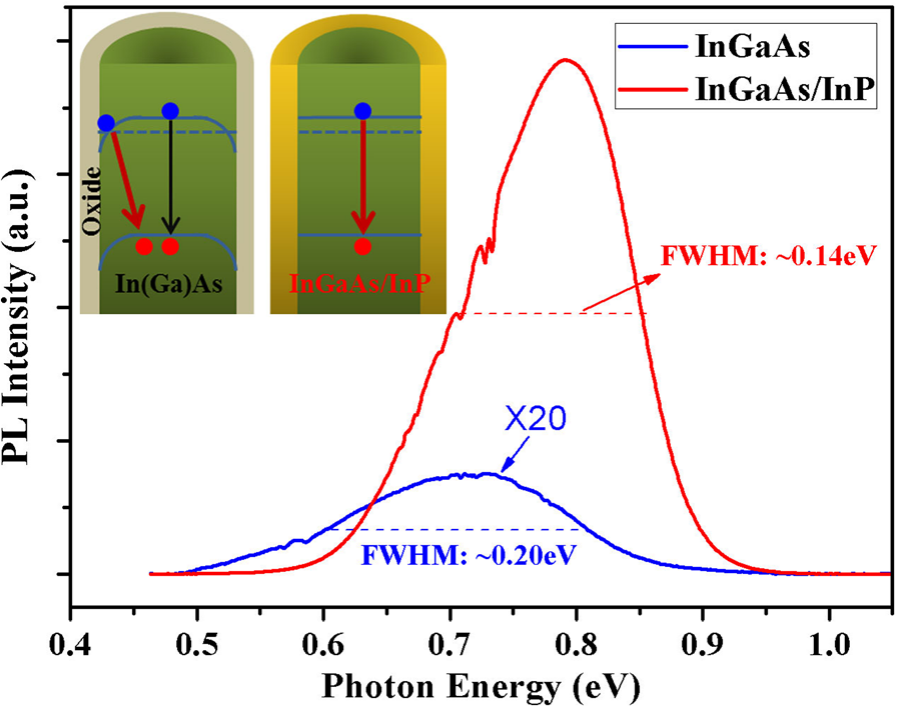
PL spectra of bare InGaAs and InGaAs/InP (Xv = 30%) core–shell nanowires at 77 K. Inset is schematic illustration of the band structures of bare In-rich InGaAs and InGaAs/InP core–shell nanowires

To investigate the optical properties of the grown nanowires, photoluminescence (PL) measurements were performed. Figure 5 compares typical PL spectra of the bare InGaAs and InGaAs/InP (Xv = 30%) core–shell nanowires at 77 K. The PL spectrum of the bare InGaAs nanowires shows a much weaker emission peaked at ∼ 0.73 eV (blue line in Fig. 5), while, the PL spectrum of the InGaAs/InP core−shell nanowires exhibits a very strong emission peaked at ∼ 0.78 eV (red line in Fig. 5) and the PL peak intensity shows a ∼ 100–fold enhancement compared to the bare InGaAs nanowires. Because the nanowire densities from different samples are comparable, we consider that such a dramatical PL emission enhancement of InGaAs/InP core−shell nanowires is caused by the effective suppression of surface states and carrier confinement by InP coating layer.

Another interesting feature is the slight blue shift of the InGaAs/InP PL peak (∼ 50 meV) compared to the bare InGaAs nanowires. First, we ascribe this different behavior to a change in the dominant carrier recombination path when the InGaAs core is coated with InP shell. Typically, for the bare InAs or In-rich InGaAs nanowires with native oxide-covered surfaces, the surface Fermi level is pinned in the conduction band induced by the numerous surface states, which will cause a downward band bending near the nanowire surface. Then, this non-flat band structure will lead to carrier redistribution where electrons accumulate near the nanowire surface while holes rather stay in the center of the nanowire. Under illumination, spatially indirect electron-hole pair transition with lower energy will be preferred, as shown in the inset in Fig. 5. For bare InAs nanowires, it has been reported that the energy difference between near band-edge emission and surface-related emission is approximately ~ 35–45 meV [21]. However, for InGaAs nanowires, because the surface band bending is significantly reduced with increasing Ga composition, this energy difference would decrease simultaneously, and then electrons are less confined near the nanowire surface and holes are less localized at the nanowire center. Therefore, we consider that the PL spectrum of the bare InGaAs nanowires is a mixing of surface-related emission and near band-edge emission. Because of the spatial separation, the surface-mediated transition probability is very low. In addition, the numerous surface states may consume extra electron-hole pairs through non-radiative recombination process. Thus, the PL intensity of the bare InGaAs nanowires is extremely weak.

However, the situation would change when InGaAs core nanowires are coated with InP shell. Because surface states of core nanowires are removed effectively and the InP shell acts as an energy barrier effectively confining carriers to the InGaAs nanowires, direct transition near band−edge with higher transition probability become dominant, as confirmed by the significant enhancement of PL emission. Moreover, due to the elimination of surface-related emission, the PL spectrum from InGaAs/InP core−shell nanowires shows a narrower full width at half maximum (FWHM) compared to the bare InGaAs nanowires. As previously mentioned, because of the alleviated surface band bending for InGaAs nanowires obtained here, the energy difference between near band-edge emission and surface-related emission should be far lower than ~ 50 meV obtained here. Thus, apart from this effect, we speculate that strain is the main origin for the observed blue-shift. Because the InP shell coherently grew on the InGaAs core free of misfit dislocations at the interface, the InGaAs core is under compressive strain, which can induce band-gap broadening of InGaAs core nanowire and account for the blue-shift of the PL peak emission [32, 33]. Therefore, by growing InP coating layer, the PL peak energy from InGaAs nanowires should display a blue-shift and its PL emission intensity can be improved greatly.

**Fig. 6:**
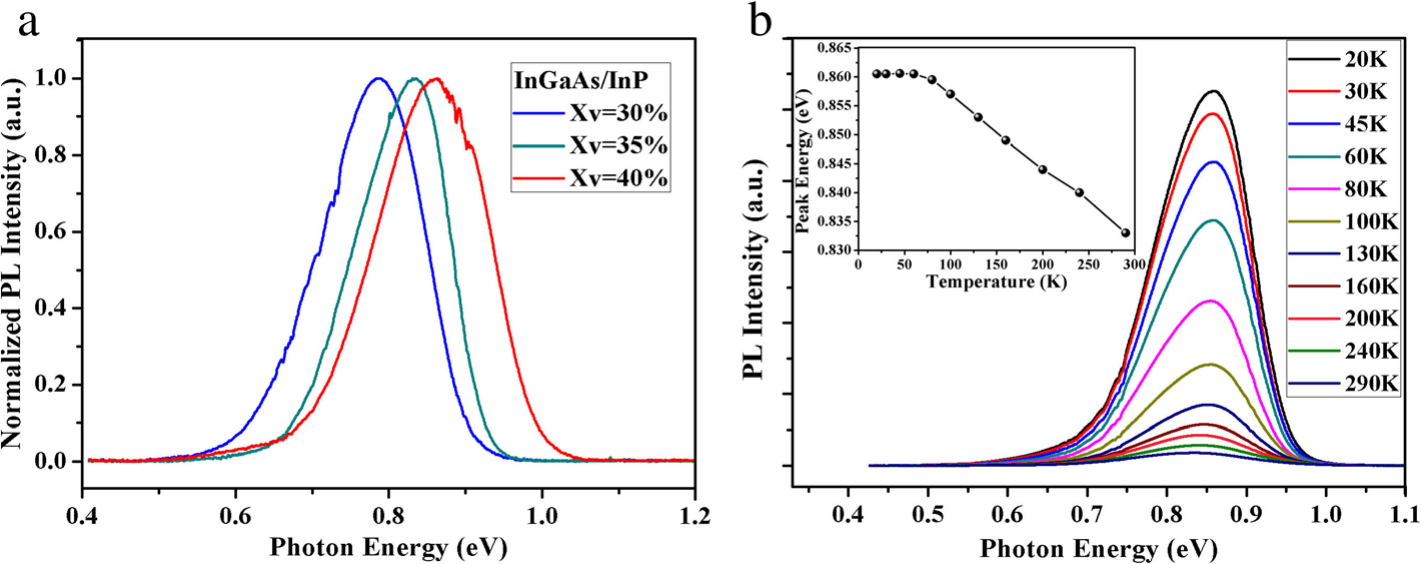
**a** Normalized PL spectra of InGaAs/InP core−shell nanowires with different Xv (Xv = 30%, 35%, and 40%) at 77 K. **b** Temperature dependent PL spectra of InGaAs/InP core−shell nanowires with Xv = 40%. Inset in **b** shows corresponding temperature dependent shift in PL peak energy

Figure 6a shows normalized PL spectra of InGaAs/InP core−shell nanowires with different Xv at 77 K. PL peak energy shows continuous blue shift (from ~ 0.78 eV to ~ 0.86 eV) with the increase of Xv in the range of 30 to 40%. Moreover, from the PL measurements at room temperature, the emission of the InGaAs/InP core−shell nanowires peaks at the wavelength range of 1.49–1.68 μm, which has minimal power attenuation in optical fiber communication (~ 1.55 μm region). Figure 6b displays temperature dependent PL spectra for the InGaAs/InP core−shell nanowire sample with Xv = 40%, and the inset shows corresponding temperature dependent shift in PL peak energy. Usually, in single-crystalline bulk material, the temperature dependence of the luminescence displays continuous red-shift with the increase of temperature according to Varshni equation. Interestingly, from the inset in Fig. 6b, the red-shift can be only observed in the temperature range of 60–290 K. When the temperature is below 60 K, PL peak energy keeps almost unchanged. Considering the high density of structural defects in the grown nanowires, we speculate that this phenomenon is most likely caused by localized trap states near the band edge [34]. At low temperature, the emission is dominated by trap-assisted. With the increase of temperature, the trapped carriers are excited from the lower-energy trap states to the band edge. Therefore, PL peak energy at low temperature region does not follow the commonly observed continuous red-shift with temperature and tends to be underestimated compared to the precise band edge.

## Conclusions

In summary, we have presented the growth and characterization of InGaAs/InP core–shell nanowires on Si–(111) substrates using MOCVD. The stress at the core–shell interface caused by the large lattice mismatch between the core and shell materials has strong influence on the growth behavior of the InP shell, leading to the asymmetric growth of InP shell around the InGaAs core and even to the bending of the nanowires. TEM measurements revealed that the InP shell coherently grew on the InGaAs core without any misfit dislocations. From the PL measurements at 77 K, the PL peak intensity of InGaAs/InP core−shell nanowires shows a ∼ 100 times improvement compared to the bare InGaAs nanowires due to the passivation of surface states and effective carrier confinement by the InP coating layer. Such significant emission enhancement of the InP-capped nanowires allows us to observe emission even at room temperature. Overall, the results obtained here further our understanding of the growth behavior of strained core–shell heterostructure nanowires and may pave the foundation for the fabrication of the optoelectronic devices based on InGaAs nanowires.

## Abbreviations

BF: Bright−field
CMOS: Complementary metal-oxide-semiconductor
EDS: Energy dispersive spectroscopy
FTIR: Fourier transform infrared
FWHM: Full width at half maximum
LED: Light-emitting diode
MOCVD: Metal-organic chemical vapor deposition
PL: Photoluminescence
SAED: Selected area electron diffraction
SEM: Scanning electron microscopy
TEM: Transmission electron microscopy
TMGa: Trimethylgallium
TMIn: Trimethylindium
ZB: Zinc-blende
